# Supplementary material for: Long-term antigen exposure irreversibly modifies metabolic requirements for T cell function
Source: eLife. 2018 Jun 18;7:e30938. doi: 10.7554/eLife.30938 (PMC6025959; doi:10.7554/eLife.30938)
Supplement: Supplementary file 2. — The figure presents examples of protein separation obtained in this study. [file elife-30938-supp2.pdf]

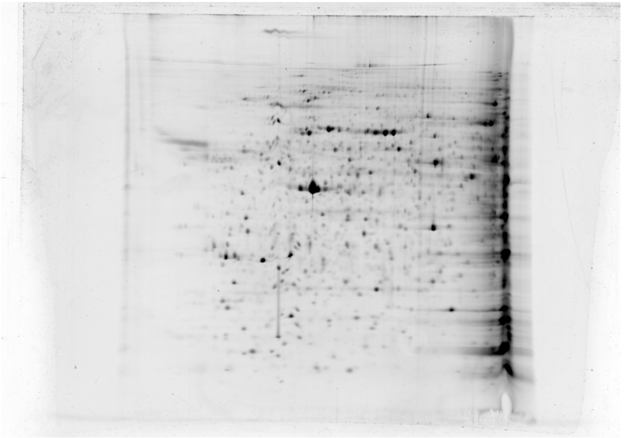

Naive

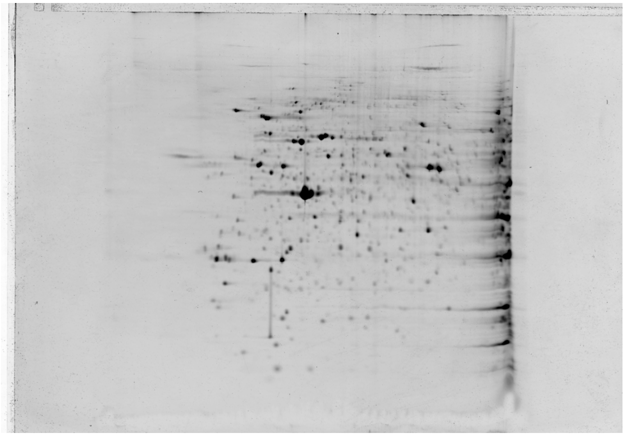

Chronic

Supplementary File 2. Two-dimensional gel electrophoresis of proteins from purified naive and chronic CD4 T cells. The figure presents examples of protein separation obtained in this study.
